# Long-Term Survival of a Patient with Invasive Signet-Ring Cell Carcinoma of the Urinary Bladder Managed by Combined S-1 and Cisplatin Adjuvant Chemotherapy

**DOI:** 10.1155/2013/915874

**Published:** 2013-05-08

**Authors:** Takashi Hamakawa, Yoshiyuki Kojima, Taku Naiki, Yasue Kubota, Takahiro Yasui, Keiichi Tozawa, Yutaro Hayashi, Kenjiro Kohri

**Affiliations:** Department of Nephro-Urology, Nagoya City University Graduate School of Medical Sciences, 1 Kawasumi, Mizuho-chou, Mizuho-ku, Nagoya 467-8601, Japan

## Abstract

Primary signet-ring cell carcinoma of the urinary bladder is extremely rare and patient survival is very poor. The disease usually presents at advanced stages because the cancer progresses rapidly. The only option for effective treatment is radical cystectomy, and no effective chemotherapy has been established for this variant. We report a case of signet-ring cell carcinoma of the urinary bladder with a long-term survival of 90 months owing to radical cystectomy and combination adjuvant chemotherapy with S-1 and cisplatin. To our knowledge, this is the first report to demonstrate the long-term therapeutic activity of combination S-1 and cisplatin adjuvant chemotherapy against invasive signet-ring cell carcinoma of the urinary bladder.

## 1. Introduction

Adenocarcinoma of the urinary bladder accounts for 0.5%–2% of all bladder cancers with signet-ring cell carcinoma being an aggressive phenotype characterized by signet-ring cells containing intracellular mucin-filled vacuole, which displaces the hyperchromatic nucleus to one side of the cell [1]. Primary signet-ring cell carcinoma of the urinary bladder (SRCCU) is a rare neoplasm that has a very high mortality rate and can be primary (arising from the bladder wall or urachus remnants) or metastatic (tumors originating in the stomach, colon, or breast) [2]. It accounts for approximately 0.24% of all bladder malignancies. This disease usually presents at advanced stages, and patient survival is therefore usually poor with a reported mean 5-year survival rate of 27%–30% [3, 4]. One quarter of the patients were found to have distant metastases at the time of diagnosis and 60% of patients died within 1 year [5]. Patients are usually treated with several types of combination chemotherapy after radical cystectomy, but survival does not improve significantly. Here we report a case of advanced SRCCU successfully treated with combination chemotherapy of S-1 and cisplatin.

## 2. Case Report

**Figure 1:**
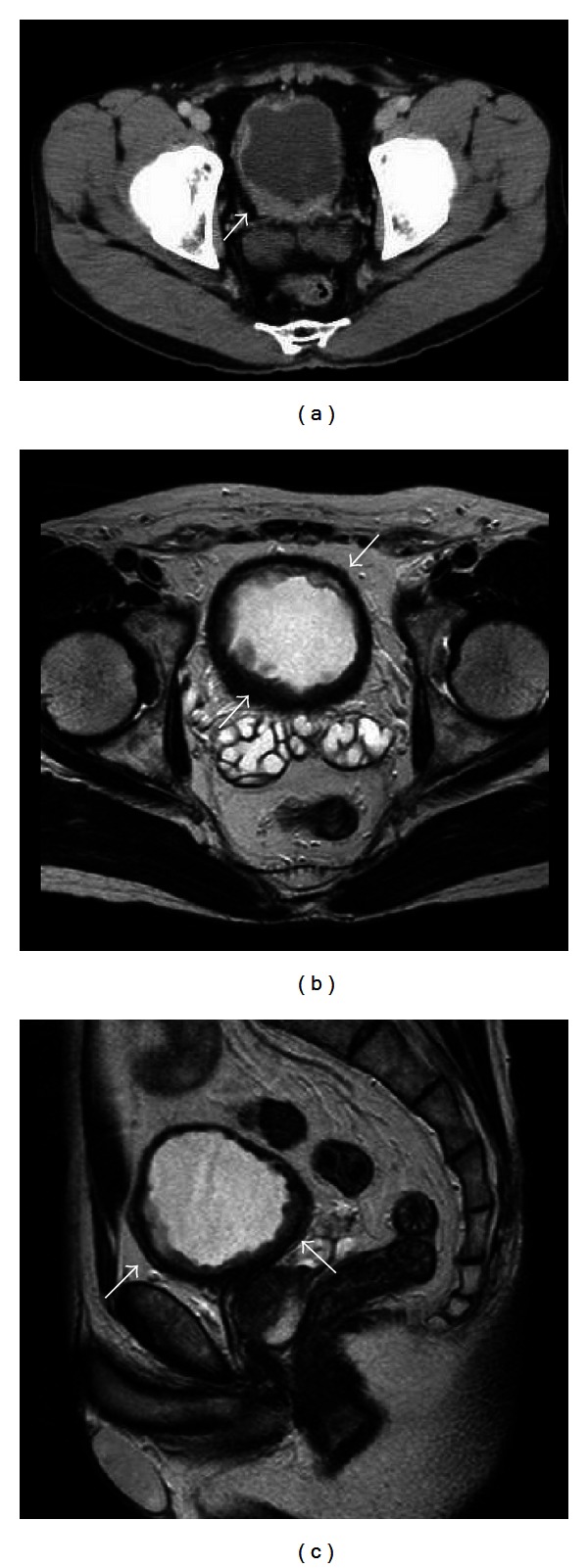
(a) CT shows diffuse tumor of the urinary bladder wall. (b)Transverse section of MRI demonstrates near-circumferential urinary bladder wall thickening. (c) Sagittal section of MRI shows urinary bladder wall thickening from anterior to posterior wall.

**Figure 2:**
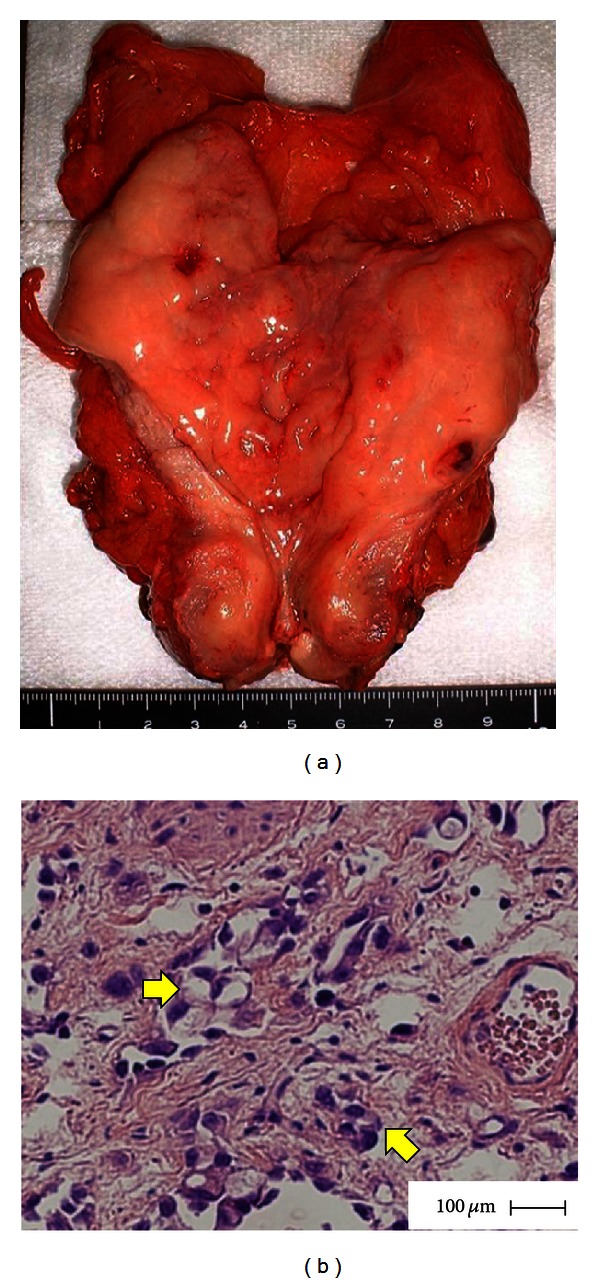
(a) Macroscopic image of resected urinary bladder specimen shows white colored, diffuse nonpapillary tumor. (b) Macroscopic appearance of the urinary bladder tumor shows poorly differentiated adenocarcinoma with multiple signet-ring cells (arrow) (H&E, high-power view).

A 53-year-old Japanese man with a history of pain on urination and hematuria for several months was referred to our hospital. He had no past medical history and was treated with a course of antibiotics for urethritis, which did not resolve the complaint. Urinalysis revealed RBC 50–99/HPF and WBC 10–19/HPF and was negative for bacteria and for urinary cytology. Serum tumor markers including carcinoembryonic antigen were within normal limits. Cystoscopy revealed widely prevalent nonpapillary raised lesions and edematous mucosa on the posterior wall of the bladder. Pelvic computed tomography (CT) scan demonstrated marked diffuse bladder wall thickening without swelling of the pelvic lymph nodes. Magnetic resonance imaging (MRI) produced results similar to CT. Moreover, sagittal MRI showed bladder wall thickening from the anterior to posterior wall (Figure 1). Transurethral biopsy of the tumor was performed and histopathological examination revealed signet-ring cell carcinoma with poorly differentiated adenocarcinoma. We then performed gastroscopy, colon fiberscopy, and radionuclide scanning evaluation to exclude an extravesical primary site. As a result, the final diagnosis was revised to SRCCU. The patient underwent radical cystectomy with pelvic lymph node dissection and the ileal neobladder. Grossly, the excised specimen had diffuse bladder wall thickening and edematous mucosa with reddened and erosive areas. Microscopically, the bladder wall showed diffuse infiltration of tumor cells, abundant mucin formation with positive staining for periodic acid-Schiff, resulting in compression of the nucleus, a characteristic signet-ring cell that had invaded the layer of a serous membrane and vessel invasion (high grade, pT3bN0M0 v(+)) (Figure 2). Adjuvant combination chemotherapy with S-1 and cisplatin was initiated in the patient because of the tumor stage and potential for micrometastasis. He received S-1 (80 mg/m^2^, days 1–14) and cisplatin (70 mg/m^2^, day 8) for three cycles. This regimen was approved by the institutional chemotherapy review board at Nagoya City University Hospital and conducted in accordance with Declaration of Helsinki. As a result, no remarkable adverse event was associated with this chemotherapy. He is still alive 90 months with no evidence of tumor recurrence or metastasis.

## 3. Discussion

SRCCU is a rare histologic variant of poorly differentiated adenocarcinoma. Most cases of SRCCU occur in middle age, and common complaints are hematuria and irritation on voiding, both of which were seen in this patient. The duration of presenting symptoms was 3 months in the review by Naeim et al. [6] and 3.5 months in the study by Blute et al. [7]. 

The histogenesis of SRCCU remains controversial. Many experts suggest that mesonephric remnants of the trigone of the bladder may be the source of this cancer. However, this would not explain signet-ring cell carcinoma at other locations. Another theory is metaplasia of urothelium, which may occur along the surface of or in areas of cystitis cystica within the bladder [13]. In this case, a microscopically similar specimen revealing urothelial metaplasia including cystitis glandularis and cystitis cystica may be due to chronic bladder inflammation. Neoplastic transformation may result in glandular adenocarcinoma or in signet-ring cells, which are produced by accumulation of mucin within their cytoplasm resulting in displacement of the nucleus to one side of the cell [14].

Radical cystectomy is the only therapy that offers the possibility of a cure when the tumor is localized. However, treatment options for advanced and invasive SRCCU have not been well defined because of the rarity of the tumor. Although combinations of methotrexate, vinblastine, doxorubicin, and cisplatin or gemcitabine and cisplatin are standard chemotherapy regimens for the treatment of advanced and invasive urothelial carcinoma, SRCCU is generally resistant to these therapies that have several limitations. Long-term follow-up has shown considerable toxicity, including myelosuppression and renal toxicity. To date, some patients fail to respond to standard platinum-based regimens but derive a significant clinical benefit from colon cancer type regimens such as 5-fluorouracil- (5-FU-) based regimens of capecitabine. In addition, some reports have shown the effectiveness of combination chemotherapy with 5-FU and cisplatin in SRCCU (Table 1). 

In Japan, S-1 is the most widely used 5-FU-based drug for the treatment of gastric carcinoma including signet-ring cell carcinoma. It is an oral 5-FU derivative consisting of tegafur, gimeracil, and oteracil. Gimeracil (5-chloro-2,4-dihydropyrimidine) is a dihydropyrimidine dehydrogenase inhibitor that inhibits degradation of 5-FU. Oteracil (monopotassium 1,2,3,4-tetrahydro-2,4-dioxo- 1,3,5-triazine-6 carboxylate) reduces gastrointestinal toxicity caused by phosphorylating 5-FU [15–17]. Administration of S-1 alone showed a high response rate of approximately 50% and low toxicity in several clinical studies of gastric cancer [18]. In addition, Sakuramoto et al. [19] reported that S-1 administration was an effective adjuvant treatment for locally advanced gastric cancer. Koizumi et al. [20] reported that median survival was significantly longer in patients with advanced gastric carcinoma who were administered S-1 and cisplatin than in those administered S-1 alone. One mechanism for the activity of this combination is that cisplatin inhibits the incorporation of exogenous L-methionine into cancer cells and increases the level of reduced folates, which are essential cofactors for the formation of a tight ternary complex of thymidylate synthase and 5-fluoro-2′-deoxyuridine-5′-monophosphatase. This results in the enhancement of the anticancer activity of 5-FU [21]. Furthermore, we previously reported a case of recurrent advanced poorly differentiated adenocarcinoma originating from urachus remnants that was treated successfully with S-1 and cisplatin [22], suggesting that this combination chemotherapy is a potent tool for controlling advanced bladder adenocarcinoma. Based on these reports, we successfully used this combination in our patient with SRCCU, resulting in long-time disease-free survival, good quality of life, and preserved renal function.

We consider the 5-FU-based chemotherapeutic regimen with cisplatin used in gastric cancer as an effective therapy for SRCCU. Our conclusion is limited by the fact that it is based on only one case; however, adjuvant chemotherapy with S-1 and cisplatin can be considered as an option for patients with invasive SRCCU. 

## Figures and Tables

**Table 1:** Description of all cases of adjuvant chemotherapy regimens used in advanced SRCCU in Japan with more than 6-month followup.

Reference	Pathological T stage	Chemotherapy regimen	Followup
Tsumatani et al. [8]	pT3b	UFT, ADM, MMC, OK432	29 months
Amemiya et al. [9]	pT3b	UFT, CDDP, ADM, CPA	Died 12 months after diagnosis
Tukumo et al. [10]	pT4	CDDP, MTX, VP-16	Died 7 months after diagnosis
Nanpo et al. [11]	pT3a	5-FU, MTX	Died 8 months after diagnosis
Fujita et al. [12]	pT3a	S-1	8 months
Our case	pT3b	S-1, CDDP	90 months

OK432: Picibanil; 5-FU: 5-fluorouracil; CDDP: cisplatin; MTX: methotrexate; UFT: tegafur + uracil; VP-16: etoposide; ADM: adriamycin; CPA: cyclophosphamide; MMC: mitomycin C; S-1: tegafur-gimeracil-oteracil potassium.

## References

[1] Grignon DJ, Ro JY, Ayala AG, Johnson DE (1991). Primary signet-ring cell carcinoma of the urinary bladder. *The American Journal of Clinical Pathology*.

[2] Jakse G, Schneider HM, Jacobi GH (1978). Urachal signet-ring cell carcinoma, a rare variant of vesical adenocarcinoma: incidence and pathological criteria. *Journal of Urology*.

[3] Romics I, Székely E, Szendroi A (2008). Signet-ring cell carcinoma arising from the urinary bladder. *The Canadian Journal of Urology*.

[4] Akamatsu S, Takahashi A, Ito M, Ogura K (2010). Primary signet-ring cell carcinoma of the urinary bladder. *Urology*.

[5] Fiter L, Gimeno F, Martin L, Gomez Tejeda L (1993). Signet-ring cell adenocarcinoma of bladder. *Urology*.

[6] Naeim F, Schlezinger RM, de la Maza LM (1972). Primary signet ring cell carcinoma of the bladder: report of a case and review of the literature. *Journal of Urology*.

[7] Blute ML, Engen DE, Travis WD, Kvols LK, Prout GR (1989). Primary signet ring cell adenocarcinoma of the bladder. *Journal of Urology*.

[8] Tsumatani K, Arai K, Kurooka K (1988). Primary signet-ring cell carcinoma of the urinary bladder: report of a case. *Hinyokika Kiyo*.

[9] Amemiya H, Iwasaki A, Hata R (1990). Primary signet-ring cell carcinoma of the urinary bladder: a case report. *Nishinihon Journal of Urology*.

[10] Tsukumo Y, Sakamoto A (2005). Signet ring cell carcinoma of the urinary bladder detected by urine cytology: a case report. *Japanese Journal of Diagnostic Pathology*.

[11] Nanpo Y, Ito K, Kitagawa M (2005). Primary signet-ring cell carcinoma of the urinary bladder with elevated serum carcinoembryonic antigen and carbohydrate antigen 19-9: a case report. *Japanese Journal of Urological Surgery*.

[12] Fujita M, Otoshi T, Kobayashi K (2009). Primary signet-ring cell carcinoma of the urinary bladder: a case report. *Hinyokika Kiyo*.

[13] Braun EV, Ali M, Fayemi AO, Beaugard E (1981). Primary signet-ring cell carcinoma of the urinary bladder: review of the literature and report of a case. *Cancer*.

[14] Bernstein SA, Reuter VE, Carroll PR, Whitmore WF (1988). Primary signet-ring cell carcinoma of urinary bladder. *Urology*.

[15] Harris BE, Song R, Soong SJ, Diasio RB (1990). Relationship between dihydropyrimidine dehydrogenase activity and plasma 5-fluorouracil levels with evidence for circadian variation of enzyme activity and plasma drug levels in cancer patients receiving 5-fluorouracil by protracted continuous infusion. *Cancer Research*.

[16] Shirasaka T, Shimamato Y, Ohshimo H (1996). Development of a novel form of an oral 5-fluorouracil derivative (S-1) directed to the potentiation of the tumor selective cytotoxicity of 5-fluorouracil by two biochemical modulators. *Anti-Cancer Drugs*.

[17] Shirasaka T, Shimamoto Y, Fukushima M (1993). Inhibition by oxonic acid of gastrointestinal toxicity of 5-fluorouracil without loss of its antitumor activity in rats. *Cancer Research*.

[18] Sugimachi K, Maehara Y, Horikoshi N (1999). An early phase II study of oral S-1, a newly developed 5-fluorouracil derivative for advanced and recurrent gastrointestinal cancers. *Oncology*.

[19] Sakuramoto S, Sasako M, Yamaguchi T (2007). Adjuvant chemotherapy for gastric cancer with S-1, an oral fluoropyrimidine. *The New England Journal of Medicine*.

[20] Koizumi W, Narahara H, Hara T (2008). S-1 plus cisplatin versus S-1 alone for first-line treatment of advanced gastric cancer (SPIRITS trial): a phase III trial. *The Lancet Oncology*.

[21] Kojima Y, Yamada Y, Kamisawa H, Sasaki S, Hayashi Y, Kohri K (2006). Complete response of a recurrent advanced urachal carcinoma treated by S-1/cisplatin combination chemotherapy. *International Journal of Urology*.

[22] Shirasaka T, Shimamoto Y, Ohshimo H, Saito H, Fukushima M (1993). Metabolic basis of the synergistic antitumor activities of 5-fluorouracil and cisplatin in rodent tumor models in vivo. *Cancer Chemotherapy and Pharmacology*.

